# Mechanical Response of Neural Cells to Physiologically Relevant Stiffness Gradients

**DOI:** 10.1002/adhm.201901036

**Published:** 2019-12-02

**Authors:** Céline Kayal, Emad Moeendarbary, Rebecca J. Shipley, James B. Phillips

**Affiliations:** ^1^ UCL Mechanical Engineering University College London Torrington Place London WC1E 7JE UK; ^2^ UCL School of Pharmacy University College London Brunswick Square, Bloomsbury London WC1N 1AX UK; ^3^ UCL Centre for Nerve Engineering University College London London WC1E 6BT UK; ^4^ Department of Biological Engineering Massachusetts Institute of Technology Cambridge MA 02142 USA

**Keywords:** mechanosensitivity, neurites, peripheral nervous system, stiffness gradients

## Abstract

Understanding the influence of the mechanical environment on neurite behavior is crucial in the development of peripheral nerve repair solutions, and could help tissue engineers to direct and guide regeneration. In this study, a new protocol to fabricate physiologically relevant hydrogel substrates with controlled mechanical cues is proposed. These hydrogels allow the analysis of the relative effects of both the absolute stiffness value and the local stiffness gradient on neural cell behavior, particularly for low stiffness values (1–2 kPa). NG108‐15 neural cell behavior is studied using well‐characterized collagen gradient substrates with stiffness values ranging from 1 to 10 kPa and gradient slopes of either 0.84 or 7.9 kPa mm^−1^. It is found that cell orientation is influenced by specific combinations of stiffness value and stiffness gradient. The results highlight the importance of considering the type of hydrogel as well as both the absolute value of the stiffness and the steepness of its gradient, thus introducing a new framework for the development of tissue engineered scaffolds and the study of substrate stiffness.

## Graphical abstract

A new protocol to fabricate a reproducible and mechanically characterized collagen based model of stiffness gradients, valuable for the study of durotaxis, is demonstrated. Morphological responses of neural cells under defined stiffness gradient profiles are investigated and for the first time the combined influence of both local stiffness magnitude and the gradient steepness is highlighted. This contributes significantly to the understanding of neural cell behavior and will underpin future improvements to nerve repair strategies.

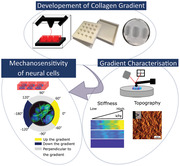

## 1 Introduction

Large gap peripheral nerve injuries are highly debilitating, causing paralysis and loss of sensation. The current clinical solution, the nerve graft, is often unsatisfactory^1^ in terms of functional recovery and has various limitations such as donor site morbidity and limited availability.^2^ Improved peripheral nerve repair strategies need to be developed and one of the emerging research avenues in tissue engineering is the study of cell–substrate interactions, which underpin conduits that support and guide neuronal regeneration. Recent studies have shown the importance of the mechanical environment within the peripheral nervous system during development^3^, ^4^ and in pathological situations.^3^, ^5^, ^6^

The interaction between the tissue and the cells is a key factor in controlling the fate of the latter.^7^, ^8^, ^9^, ^10^, ^11^, ^12^ Cells exert forces on, and sense the stiffness of the surrounding extracellular matrix (ECM).^12^ The mechanical properties of the microenvironment have important implications in cell differentiation,^13^, ^14^ proliferation,^15^ and migration.^16^, ^17^ For nervous system cells, neurite outgrowth and branching patterns have been shown to depend on the substrate stiffness.^4^, ^8^, ^18^, ^19^ Moreover, in the peripheral nervous system (PNS)^20^, ^21^ and central nervous system (CNS)^8^, ^22^ native tissues are mechanically heterogeneous; therefore, neurites are likely to encounter regions with distinct mechanical properties. Although several studies have shown the importance of chemotactic, haptotactic, and topographical guidance cues, there is still limited understanding of how mechanical cues influence neurite outgrowth and branching patterns. Recent studies have characterized the mechanosensitivity of PNS neural cells on various stiffness substrates. For example, Rosso et al.^4^ investigated the behavior of dorsal root ganglion (DRG) explants when exposed to 1, 10, and 20 kPa substrates and observed variations in the extension pattern and directionality, dependent on the substrate stiffness. Furthermore, mechanical heterogeneity encountered in nervous tissues, both CNS^8^ and PNS,^20^ raised interest in investigating the impact of stiffness gradients. Koser et al.^8^ have highlighted cellular durotactic responses to a stiffness gradient, by showing that axon bundles were orientated toward the softer side of their growth substrate. Therefore, biomimetic mechanical gradients have the potential to be used in the construction of nerve repair materials, to improve axon pathfinding and accelerate nerve repair by controlling the orientation of neurite regeneration.^4^, ^8^, ^17^, ^23^

In order to inform the development of appropriate substrates, key information about the effect of local stiffness gradients both in terms of the base stiffness value and steepness of the gradient needs to be obtained. In this study, model substrates were developed using collagen hydrogels in which defined gradients of stiffness were generated and characterized. Collagen type I is the major ECM component of the PNS and supports cellular nerve function from development to adulthood.^24^ It is also known as a suitable cell substrate for use in nerve tissue engineering.^23^, ^25^, ^26^, ^27^, ^28^, ^29^ A variety of methods exist to modify the mechanical properties of collagen, e.g., crosslinking collagen using enzymes or irradiation,^23^, ^30^, ^31^, ^32^ or blending collagen gels with other materials.^32^, ^33^, ^34^ However, these techniques alter the structure of the matrix and can create other undesirable signaling cues and variables that prevent dissecting the influence of material gradients on neurite behavior. Thus, here, we have adapted tissue engineering technology for generating gradients within RAFT‐stabilized collagen gels^35^, ^36^ by designing and 3D printing moulds to yield model collagen hydrogels with defined stiffness gradients. We used these collagen constructs to explore the influence of stiffness gradient and magnitude on neuronal cell elongation and orientation in vitro.

## 2 Experimental Section

### 2.1 Gradient Computer‐Aided Design (CAD)

**Figure 1:**
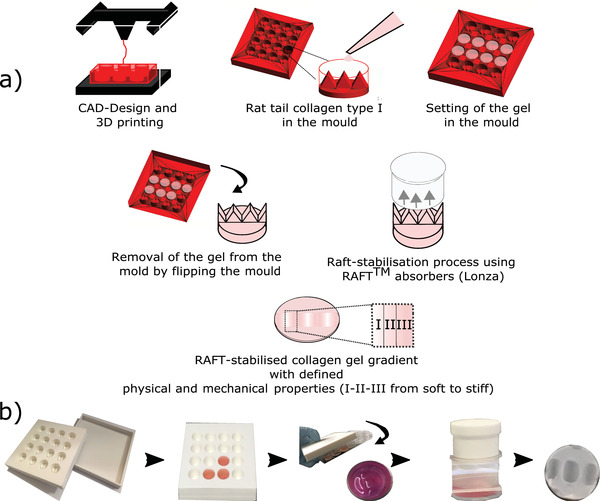
a) Schematic diagram showing the fabrication process of the collagen gradient gel mould with defined geometry were CAD‐designed and 3D printed using PLA. Collagen gel solution was pipetted in moulds and gels were left to set for 15 min in a humidified incubator and then flipped to a standard 24‐well plate. Then, gels were RAFT‐stabilized for 15 min. The gels are segmented into three separate regions (I–II–III) from soft to stiff. b) Photographs of the experimental setup to make gradients gels.

For this study, gradient moulds were designed using the CAD software AutoCAD, and 3D printed from polylactic acid (PLA) using an Ultimaker 2. Two different multiwell moulds were produced, each of which contained wells of the same dimensions as a standard 24‐well plate and therefore could be used to cast multiple equivalent collagen hydrogels simultaneously. The bottom surface of each mould well was shaped to include 3× equally spaced raised ridges, conferring a pattern of varying depth on the collagen hydrogels cast in the moulds subsequently (**Figure** **1**). One of the mould designs had ridges of height 5 mm to create a shallow gradient, referred to as “Lower;” the other had ridges of height 8 mm to create a steeper gradient, referred to as “Higher.” Moulds were sterilized by immersion in 70% ethanol overnight.

### 2.2 Raft‐Stabilized Collagen Gel Gradients Substrate Fabrication

Acid‐solubilized type I collagen from rat tail tendon (2 mg mL^−1^ in 0.6% acetic acid; First Link, UK) was mixed at a ratio of 80%: 10 v/v% with 10 × Minimum Essential Medium (MEM), 5 v/v% of Dulbecco's modified Eagle's medium (DMEM), and was neutralized with 5 v/v% of 0.325 m NaOH to achieve pH 7.4.^37^, ^38^ A volume of 1.4 mL of the collagen solution was pipetted into the printed moulds (Lower and Higher) and into a standard 24‐well plate for the control (Control), and kept in a humidified cell culture incubator (37 °C, 5% CO_2_) for 10 min to allow gelation.

Once set, gels were flipped from the printed moulds into a standard 24‐well plate and were RAFT‐stabilized for 15 min using RAFT absorbers (Startorius Stedim/Lonza). This step rapidly removed most of the fluid of the hydrogel through the top surface of the gel and resulted in gels with a flat top and bottom surface, but with gradients of collagen density resulting from the variation in gel height as a consequence of the ridges in the original casting mould (Figure 1). Gels were covered with serum free DMEM and kept in a humidified incubator for 1 h before seeding the cells.

### 2.3 Physical Characterization of Collagen Substrates

#### 2.3.1 Density of Collagen for Stabilized Gradient Gels

Stabilized gel heights were measured using an optical contact angle meter (OCAM) (KSV's CAM 2000). The images were extracted and analyszed on ImageJ. For accuracy, three measurements of the height of each gel were taken and averaged. Using the height measured, the volume post stabilization (*V*_ps_ in mL) and the mass of collagen (*m*_c_ in mg) was calculated for each of the three segmented regions (I–II–III, Figure 1a). The collagen density for each gradient segment was then defined as follows, $\rho_{\mathrm{coll}}=\frac{m_{c}}{V_{\mathrm{ps}}}$, where ρ_coll_ (mg mL^−1^) is the density of collagen.

### 2.4 Atomic Force Microscopy (AFM) Topography

A JPK Nanowizard 1 AFM (JPK Instruments Ltd., Germany) was used to obtain 10 × 10 µm high‐resolution topography images (1024 × 1024 pixels) from each segment (I–II–III, Figure 1a) of the Lower and Higher gradient gels and the Control gel. Gels were kept for 24 h in phosphate buffered saline (PBS) and rinsed using distilled water. Imaging was conducted using MSNL‐10‐A tip cantilevers (Bruker Ltd., France) with the following characteristics: resonant frequency of ≈22 kHz and spring constant ≈0.07 N m^−1^. JPK/SPM data processing software was used to extract data.

### 2.5 Mechanical Characterization of Collagen Substrates

In order to quantify the effect of collagen concentration on mechanical properties of the material, a series of force mapping experiments were performed to yield a stiffness map using AFM‐force spectroscopy. Indentation measurements were performed using a JPK Nanowizard CellHesion 200 with motorized precision stage (JPK Instruments AG, Berlin, Germany). Glass microspheres (S‐SLGMS, diameter 50–53 µm, Cospheric, Santa Barbara, CA) were glued (light cure adhesive, Loctite 349 IMPRUV, R. S. Hughes Company, Plymouth, MI) to nonconductive silicon nitride and triangular tip‐less cantilevers (NP‐O10, Brukers, spring constant ≈0.35 N m^−1^). Experiments were carried out using force mapping over 1 × 2.5 mm area, covering the segments I–II–III, at room temperature. The collected force curves were batch‐analyzed using JPK/SPM data processing software (JPK Instruments Ltd., Germany). A Hertz model was used for determining the Young's Modulus *E* (kPa), assuming a Poisson ration (ν) of 0.5.^39^

### 2.6 Neural Cell Culture on Stiffness Gradient Gels

#### 2.6.1 NG108‐15 Neural Cell Culture on Stiffness Gradient Gels

NG108‐15 (mouse neuroblastoma × rat glioma hybrid, HPA Culture Collections) were trypsinized and 1 × 10^5^ cells per gel were seeded onto RAFT‐stabilized collagen gradient gels (Lower and Higher gradient gels, and Control gel with no gradient) contained in 24‐well plates, in serum‐free cell culture medium.^40^ Cultures were maintained for two days (*t* = 48 h) in a humidified incubator (5% CO_2_ at 37°).

### 2.7 Cell Immunolabeling and Fluorescence Imaging

Cultures were fixed with 4% paraformaldehyde overnight at 4 °C and permeabilized using 0.5% Triton X‐100 for 30 min. Nonspecific binding was blocked with 5% normal goat serum. Nuclei were labeled using Hoechst (1:1000). Mouse anti‐β‐III‐tubulin primary antibody (1:400, incubated overnight at 4 °C) and dylight goat antimouse 488 (1:300, for 90 min) were used to detect the neurites. Samples were rinsed using PBS) between each step. Fluorescence microscopy (Zeiss Axio Lab.A1, Germany) was used to acquire images of NG108‐15 cells on the gels. Images were captured using a 10× lens, and were analyzed using ImageJ software (US National Institutes of Health).^41^

### 2.8 Data Analysis

Neurite response to collagen gradients was quantified using established measures^42^ including the number of cells forming neurites; the mean number of neurites per cell; the mean neurite length per cell. Neurite length was determined using the freehand line selection tool in ImageJ and measure function (Figure 4a), and the angle of each neurite was measured from the long axis of the gradient (Figure 4a). Cell branching was considered if at least one of the neurites was branched. Orientation was classified into three different categories: a) neurites elongating up the gradient, toward stiffer substrate region ([−60°;60°]), b) neurites elongating down the gradient, toward softer substrate region ([−120°;120°]), and c) neurites elongating perpendicular to the gradient. The categories are visualized in Figure 4a.

### 2.9 Statistical Analysis

For the mechanical and in vitro experiments, statistical analysis was performed using JMP Pro 13 software (JMP SAS Institute, Marlow, Buckinghamshire, UK). Data are presented as mean values ± standard deviation (SD). A Shapiro–Wilk test was used to test the normality of the distributions. The differences between the multiple groups were evaluated by performing a one‐way ANOVA test followed by a Tukey–Kramer post hoc test to obtain the multiple comparison *P* values. Statistical significance was taken at *P* < 0.05.

## 3 Results

### 3.1 Gradient Characterization

**Figure 2:**
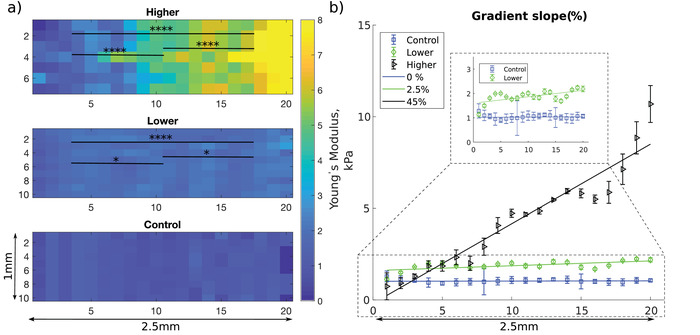
a) Spatial mapping of the elastic modulus of Control gel and Lower and Higher gradient gels. The elastic modulus at each pixel is the mean of five measurements taken by AFM indentation, and is represented as a color map with blue denoting softer (0 kPa) and yellow corresponding to stiffer (8 kPa) regions. Stiffness distributions (*E*, kPa), for each of the three segment (I–II–III) of the Lower and Higher gels were statistically analyzed. *N* = 56 per segmented area for the Lower gradient and *N* = 49 per segmented area for the Higher gradient (one‐way ANOVA test and Tukey–Kramer multiple comparison's test). Asterisks indicate significant statistical differences (*P < 0.05, ****P < 0.0001). b) Mean Young's modulus averaged for 3 gels every 125 µm over a 2.5 mm distance (corresponding to 20 segments) and corresponding gradient slope (Control (0%), Lower (2.5%), and Higher (45%)).

We fabricated collagen substrates with stiffnesses of physiological relevance to the PNS^4^, ^5^, ^17^ by varying the collagen concentration using bespoke 3D‐printed moulds and the RAFT‐stabilization process. Two different collagen gradients (Lower and Higher) were created and compared to control plain RAFT‐stabilized gels with no gradient (Control). For Lower gradient, the collagen density ranged from 67.0 ± 4.2 to 79.0 ± 7.1 mg mL^−1^, and yielded a Young's Modulus (*E*) ranging from 1.7 ± 0.3 to 2.0 ± 0.2 kPa (**Table** **1** and **Figure** **2**). For Higher gradient, the collagen density ranged from 73.0 ± 7.1 to 122.0 ± 4.8 mg mL^−1^, and yielded an *E* value ranging from 1.5 ± 0.6 to 7.1 ± 1.9 kPa (Table 1 and Figure 2). Averaged gradient slope, estimated as change of *E* over distance, was 0.85 kPa mm^−1^ for the Lower gradient and 7.96 kPa mm^−1^ for the Higher gradient systems (Table 1 and Figure 2a). For the analysis of cell behavior (Section 4), the continuous stiffness gradients were each segmented in three areas (I–II–III). Statistical analysis showed no significant difference between the Control and the softest part of each gradient gel (I_Lower_ and I_Higher_). The three segments (I–II–III) within both Lower and Higher gradient were significantly different (Figure 2a). For the Lower gradient, the stiffness variation between the softest (1.7 ± 0.3 kPa) and the stiffest region (2.0 ± 0.2 kPa) is highly significant (*P* < 0.0001).

**Table 1:** Characteristics of RAFT‐stabilized collagen substrates, including the collagen density (mg mL^−1^) and the corresponding Young's modulus (*E*, kPa) for the Control and the segmented area (I–II–III) of the Lower and Higher gradient gels measured using AFM‐force spectroscopy, as well as the orientation of neurites. On the stiffer region of both gradient gels, more persistent directionality along the gradient is observed (bold values). Data are presented as mean ± SD

Gradient	Segment label	Slope	Density	≈*E*	Orientation
		[kPa mm^−1^]	[mg mL^−1^ ± SD]	[kPa ± SD]	[along the gradient%]
Control	–	0	70 ± 5.7	1.0 ± 0.2	60.6
	I_Lower_		67 ± 4.2	1.7 ± 0.3	60.0
Lower	II_Lower_	0.85	71 ± 5.9	1.9 ± 0.1	**70.1**
(0.85 kPa mm^−1^)	III_Lower_		79 ± 7.1	2.0 ± 0.2	**67.0**
	I_Higher_		73 ± 7.1	1.5 ± 0.6	58.7
Higher	II_Higher_	7.96	86 ± 6.9	4.1 ± 1.1	61.1
(7.96 kPa mm^−1^)	III_Higher_		122 ± 4.8	7.1 ± 1.9	**70.2**

### 3.2 Topography of Collagen Gradients

**Figure 3:**
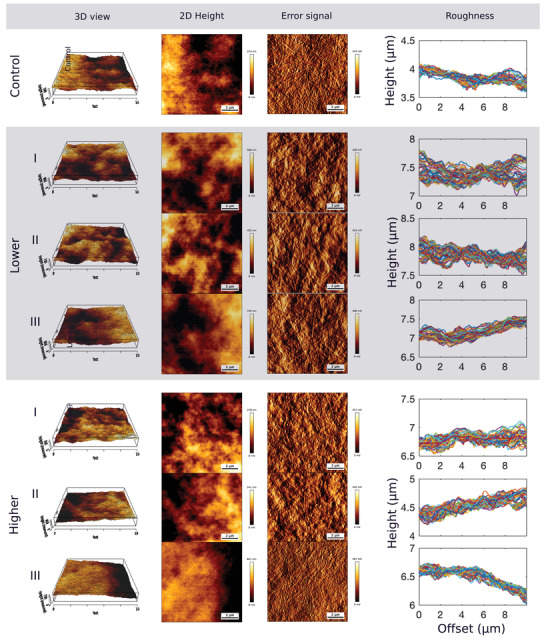
AFM topography images (10 × 10 µm, 1024 × 1024 pixels) of RAFT‐stabilized collagen gels for the Control gel, and the three segments (I–II–III) of the Lower and Higher gels. The first two columns show the 3D and 2D view of the relative value of the height of the gels (scale bar in nm). The third column is the error signal (mV) indicating the surface of the collagen gels where the banding pattern of collagen type I fibrils is visible. The surface roughness of the gels is indicated by the height profiles in the last column.

AFM imaging was used to examine the topography of the collagen gels and investigate any surface modification induced by the process of fabricating gradients. **Figure** **3** shows AFM images of three different segments I, II, and III (Table 1) for the Control, Lower, and Higher gradient gels. All three types of substrate exhibited a similar dense surface structure with distinctive collagen fibrils visible. The collagen fibrils were randomly oriented in each of three different types of substrate, and did not present any apparent topographical directional cues. The measurement of the gel height shows surface structure with less than 1 µm height and a maximum variation of 745 nm for III_Lower_, therefore the overall surface topography and roughness of the gels can be negligible and the gels can be considered as flat.

### 3.3 Mechanosensitivity of NG108‐15 Cells to the Created Gradient Gels

**Figure 4:**
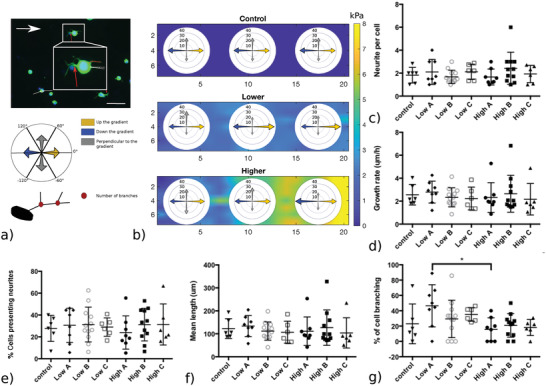
Representative fluorescence micrographs showing NG108 cells after 48 h in culture on top of gradient or Control gels. Nuclei were labeled with Hoescht (blue) and β‐III‐tubulin immunoreactivity (green) was used to detect neurites on each part (I–II–II) of the Lower and Higher gradient gels, indicated by the white arrows. White scale bars: 100 µm.

The behavior of NG108‐15 cells was evaluated on Lower (0.85 kPa mm^−1^), and Higher gradient (7.96 kPa mm^−1^) gels, for each segment area (I–II–III; Table 1), and compared to cell behavior on mechanically uniform Control gels (**Figure** **4**c–g). The number of cells presenting neurites and the number of neurites per cell were not influenced by either the absolute stiffness or the stiffness gradient of the substrate. Overall, ≈29% of cells expressed neurites (≈2 neurites per cell). The mean neurite length measured from the longest branch of each neurites was 116.4 ± 66.5 µm for the Higher gradient and 117.0 ± 42.3 µm for the Lower gradient, which was not significantly different to the 122.0 ± 60.0 µm reached by neurites on the mechanically uniform Control substrate. These data give an average neurite growth rate of 2.5 ± 1.1 µm h^−1^.

**Figure 5:**
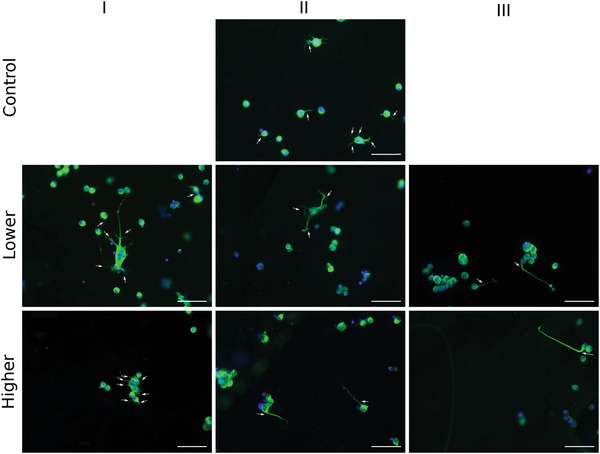
Quantification of neurite response to gradient gels. NG108‐15 cells were cultured on two different gradient slopes (Lower and Higher) and on Control gel for 48 h. a) Fluorescence micrographs show sprouting of neurites; red dashed line indicates neurite length, branching spots are indicated by red arrows, and neurite orientation was measured as indicated by the white line. The white arrow indicates the direction of the gradient. Schematic indicates the classification of neurite orientation: neurites were considered as growing up the gradient for angles between −60° and 60° (yellow), down the gradient for the angles between −120° and 120° (blue), and perpendicular to the gradient otherwise (gray). Measurements were performed for each gradient segment (I–II–III) of three separate gels, for each condition (*N* = 3, *n* = 3). b) Percentage of elongation toward a given direction for each segment of the Control and gradient gels. c) Mean number of neurites per cell, d) mean neurite growth rate (µm h^−1^), e) percentage of NG108‐15 cells presenting neurites, and f) mean neurite length (µm) do not vary according to the presence of a gradient or differences in absolute stiffness value. However, g) the extent of neurite branching (%) is affected by the stiffness gradient. Data are shown for the different segments (I–II–III) of each gradient type (Lower and Higher) and the Control. Results are shown as mean ± SD (one‐way ANOVA test and Tukey–Kramer Multiple's comparison's test, *P < 0.05).

Figure 4g shows that after 48 h, neurites had branched differently depending on the growth substrate region. Neurites branched ≈2 times more on the segment I_Lower_ of the Lower gradient (*E* ≈ 1.7 kPa) compared to the segment I_Higher_ of the Higher gradient (*E* ≈ 1.5 kPa), suggesting that NG108‐15 cells were responding differently to the local gradients present in these substrates which were otherwise mechanically equivalent in terms of absolute stiffness. This phenomenon can be observed **Figure** **5**.

Figure 4b and 5 show how the neurites explored their environment. On softer substrate segments (I_Lower & Higher_) and the Control, respectively, 1.7, 1.5, and 1.0 kPa (Table 1), neurites did not elongate in any preferential direction. On the stiffer region of both gradient gels, we observed a more persistent directionality along the gradient, regardless of the absolute stiffness value. For the Lower gradient, neurites on the stiffer segment (III_Lower_, *E* ≈ 2.0 kPa) elongated preferentially up the gradient, toward stiffer regions. In contrast, on the stiffer segment of the Higher gradient (III_Higher_), neurites elongated preferentially down the gradient toward the softer region. Overall, NG108‐15 cells responded to the local mechanical environment conferred by the gradient gels, altering both branching behavior and orientation.

## 4 Discussion

Our primary goal was to design a flat substrate with a characterized rigidity gradient to study cell responses to mechanical cues. We created physiologically relevant stiffness gradients within the range of modulus values reported previously for PNS tissue^4^, ^17^ using tissue engineering techniques to control collagen matrix density.^35^, ^36^ Collagen gels were generated and characterized, exhibiting a flat surface topography and continuous linear gradients of defined density, which correlated to the stiffness.^47^, ^48^ Statistical analysis of the three segment (I–II–III) within the Lower and Higher gradient gels indicated that the softest parts of each were comparable to the Control and that the stiffness of the three segments within each gradient gel were significantly different. Due to the limitation of the RAFT‐stabilization method,^49^ this study is limited to analyzing the relative effects of absolute stiffness value and the local stiffness gradient on a range of 1–2 kPa, compared to a uniform control gel (1 kPa). In addition, cell behavior on stiffer area (5–10 kPa) of the Higher gradient gels is reported.

**Figure 6:**
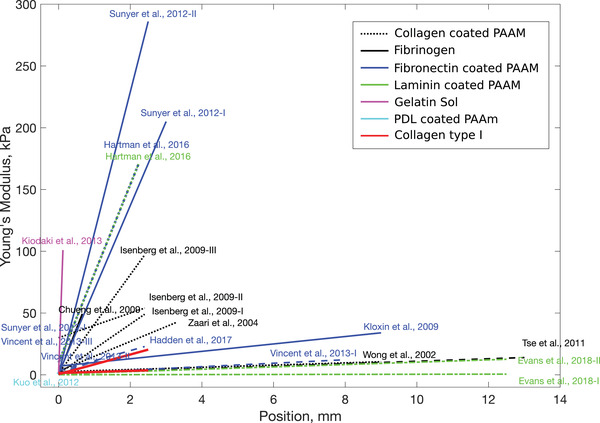
Graphical summary of the gradients and materials used to study durotactic behavior in vitro, showing the Young's Modulus (kPa) as a function of the location on the samples (mm). Studies are categorized by material used, with the majority using coated PAAm gels as it offers the possibility to work with a wide range of stiffnesses using a large variety of techniques.^14^, ^30^, ^32^, ^43^, ^44^, ^45^ This summary includes only 2D cultures. The study of durotaxis is challenging, as it is difficult to uncouple the stiffness of a substrate from its pore size, ligand molecule coating density, and height of the substrate itself.^46^ Here, we use collagen type I gels to develop defined stiffness gradients, represented by the red lines (Lower and Higher), with consistent stiffness properties to those explored in the literature.

Previous studies to create 2D substrates with continuous stiffness gradients have used coated polyacrylamide gels (PAAm) gels as well as proteins such as fibrin and gelatin. The range of stiffnesses obtained vary over several orders of magnitude^50^ and the various stiffness gradients used previously to investigate cell mechanosensitivity are shown in **Figure** **6**, categorized according to the type of material used.^14^, ^30^, ^31^, ^32^, ^43^, ^51^ Type I collagen is an appropriate choice for studying neuronal responses since it is a major component of nerve tissue. Previous studies using collagen to form gradients have tended to combine the protein with PAAm and gradients have been generated via crosslinking with enzymes, ultraviolet light, or temperature.^52^ Such crosslinking can modify the protein structure^47^, ^53^ which can potentially add confounding signaling parameters that influence cell behavior.

By contrast, the approach used here does not affect the microstructure of the collagen fibrils and the banding pattern is conserved after gel stabilization, offering a way to make gradients purely of collagen, in a simple, controlled, and reproducible way. Previous studies have reported the effects of similar substrate stiffness profiles for other types of cells (including human adipose‐derived stem cells,^54^ human mesenchymal stem cells,^14^, ^55^ vascular smooth muscle cells,^43^ fibroblasts,^50^, ^56^ and Schwann cells^17^). Focusing on the improvement of nerve repair solutions, Evans et al.,^17^ have shown that a 0.95 kPa mm^−1^ gradient induces Schwann cell durotaxis. Our Lower gradient (0.85 kPa mm^−1^) therefore could potentially be used to modulate both Schwann cells and neural cells combined for the improvement of nerve guidance conduits.

This study provides the first evidence that neurite elongation can be influenced by a combination of substrate stiffness absolute value and the steepness of the gradient slope, suggesting that neurites have the potential to process a combination of mechanical guidance cues. Neurite branching is potentially an important parameter for the establishment of synaptic connections,^18^ needed for successful regeneration.^34^, ^57^ Franze et al., 2010–2013^3^, ^58^ have shown the influence of substrate compliance on neurite branching and neurite orientation, and investigated the rigidity range with a view to understanding ECM–growth cone interactions.^4^, ^18^, ^59^, ^60^ Here, consistently, a higher percentage of cells presented branching neurites on the more compliant area of the Lower gradient (I_Lower_). A similar phenomenon was reported by Flanagan et al.^18^ and Franze et al..^3^ However, when comparing the segments with similar absolute stiffness value (Control, I_Lower_, and I_Higher_), the percentage of cells presenting branching neurites was significantly greater on the Lower gradient. This observation highlights the potential of neuronal cells to be mechanosensitive to specific stiffness gradient slopes as opposed to only the absolute stiffness value of the substrate. Rosso et al.,^4^ investigated neurite orientation on mechanically uniform substrates of different stiffnesses (1–10–20 kPa), and their results suggested that within the physiologically relevant stiffness range tested,^5^ the directionality of neurite outgrowth was substrate‐stiffness sensitive. In our study, the neurites did not orientate in a particular direction on the soft areas (Control, I_Lower_, and I_Higher_ ≈1 kPa). However, on the stiffest area of the Lower gradient gel (III_Lower_ ≈2 kPa), we observed a preferential orientation toward stiffer areas. A preferential orientation was also observed on the stiffest region (III_Higher_ ≈10 kPa) toward softer areas. It is known that the growth cone detects guidance cues and has a major role in neurite orientation.^3^, ^4^, ^58^, ^59^ This observation supports the possibility that both the absolute stiffness of the substrates and the substrate gradient slope dictate neuronal behavior. Further work will allow a better understanding of how neuronal growth cones process and convert the combination of gradient slope and absolute stiffness value into intracellular biochemical signals leading to neurite reorientation.

Neurons on different part of the stiffness gradient gels did not exhibit significant differences in neurite length, proportion of cells presenting neurites, or number of neurite per cell (substrate stiffness from 1 to 10 kPa). This result is in contrast with Rosso et al.,^61^ who noted that length of neurite outgrowth from embryonic DRG explants was sensitive to substrate stiffness (substrate stiffness between 1 and 10 kPa). In addition, Leach et al.,^62^ have shown that neurites were longer, more branched, and a greater percentage of cells presented neurites on stiff substrates (stiffness between 10^2^ and 10^4^ Pa) compared to soft substrates (10 Pa). However, in their study, no significant differences were found on substrates with a stiffness greater than 10^2^ Pa. The difference observed could potentially be explained by the difference in the cell type used for the study, the difference in the substrate composition, the absence of additional external cues (e.g., external growth factors), or the introduction of a stiffness gradient slope as previously hypothesized by Leach et al.^62^ Related work has investigated neurite growth behavior within 3D substrates with varying stiffnesses,^60^ however the focus of this study was restricted to neuronal behavior on surfaces of substrates with defined stiffness environments. Further studies would be required to separate the influence of absolute stiffness and stiffness gradient slope in order to investigate the effects on neuronal growth further.

## 5 Conclusion

This study describes a technique to fabricate type I collagen gels with controlled and reproducible physical (density) and mechanical (stiffness) gradients, without damaging collagen fibril structure nor adding additional chemical modification. These gradient gels offer a valuable platform to study cell–substrate interactions. Neural cells responded to the gradients by modifying their branching behavior and orientation. This study provides important information on neuronal growth‐cone durotaxis, demonstrating neurite orientation in response to the presence of a stiffness gradient, and revealing previously unreported sensitivity to the combination of gradient slope and absolute stiffness. Neurites grew toward stiffer or softer substrate regions, depending on the gradient/absolute stiffness combination, highlighting how both cues combine to control orientation. This work presents a promising strategy for future neural tissue engineering approaches that harness mechanical features of biomaterials to support and guide neuronal regeneration.

## Conflict of Interest

The authors declare no conflict of interest.
